# Genomic introgression through interspecific hybridization counteracts genetic bottleneck during soybean domestication

**DOI:** 10.1186/s13059-019-1631-5

**Published:** 2019-01-30

**Authors:** Xutong Wang, Liyang Chen, Jianxin Ma

**Affiliations:** Department of Agronomy, Purdue University, West Lafayette, IN 47907 USA

**Keywords:** Domestication, Genetic diversity, Introgression, Natural selection, Recurrent selection, Selective sweep, Varietal diversification

## Abstract

**Background:**

Evidence of introgression, the transfer of genetic material, between crops and their wild relatives through spontaneous hybridization and subsequent backcrossing has been documented; however, the evolutionary patterns and consequences of introgression and its influence on the processes of crop domestication and varietal diversification are poorly understood.

**Results:**

We investigate the genomic landscape and evolution of putative crop-wild-relative introgression by analyzing the nuclear and chloroplast genomes from a panel of wild (*Glycine soja*) and domesticated (*Glycine max*) soybeans. Our data suggest that naturally occurring introgression between wild and domesticated soybeans was widespread and that introgressed variation in both wild and domesticated soybeans was selected against throughout the genomes and preferentially removed from the genomic regions underlying selective sweeps and domestication quantitative trait locus (QTL). In both taxa, putative introgression was preferentially retained in recombination-repressed pericentromeric regions that exhibit lower gene densities, reflecting potential roles of recombination in purging introgression. Despite extensive removal of introgressed variation by recurrent selection for domestication-related QTL and associated genomic regions, spontaneous interspecific hybridization during soybean domestication appear to have contributed to a rapid varietal diversification with high levels of genetic diversity and asymmetric evolution between the nuclear and chloroplast genomes.

**Conclusions:**

This work reveals the evolutionary forces, patterns, and consequences of putative genomic introgression between crops and their wild relatives, and the effects of introgression on the processes of crop domestication and varietal diversification. We envision that interspecific introgression serves as an important mechanism for counteracting the reduction of genetic diversity in domesticated crops, particularly the ones under single domestication.

**Electronic supplementary material:**

The online version of this article (10.1186/s13059-019-1631-5) contains supplementary material, which is available to authorized users.

## Background

Soybean (*Glycine max* [L.] Merr.) is one of the most economically important crops in the world, providing a source of high-quality proteins for feed and food as well as vegetable oil/fuels for human consumption and industrial use [1]. It has been widely accepted that soybean was domesticated from its annual wild relative *Glycine soja* in China approximately ~ 6000–9000 years ago [2, 3], resulting in dramatic morphological and physiological modifications often referred to as the “domestication syndrome” [4]. This process was followed by varietal diversification, forming a multitude of soybean landraces adapted to diverse eco-regions for cultivation in agricultural systems. This scenario, i.e., the single origin of cultivated soybeans, appears to be well supported by recent investigation of genome-wide diversity among cultivated and wild soybeans at population levels, in which all cultivated accessions were exclusively grouped together into a single clade interior to the *G. soja* clades [5–7]. The single origin model is also supported by recent identification and isolation of a few key domestication genes such as *GmHS1-1* and *B1*, which control seed coat impermeability and seed coat bloom, respectively [8, 9]. At each of the two domestication loci, the causal mutation responsible for the domestication transition was shared by phylogenetically defined major groups of landraces that are representative of the cultivated soybean population.

Despite the general acceptance of the single-origin model, the domestication history and processes of soybean remain obscure and even under debate [10]. An early study proposed that the transition from the wild to domesticated soybeans occurred as a gradual process [11]. This proposition was solely based on the estimated time (~ 0.27 million years) of divergence between the soybean (cv., Williams 82) reference genome [12] and a sequenced *G. soja* accession (var. IT82932) [11]. Given that many wild relative of crop (WRC) accessions are highly diverged from their related crops, such as maize, of single domestication origin [13, 14], such a divergence time does not necessarily suggest the existence of artificially selected intermediates or *G. soja*/*G. max* complex from which the cultivated soybean was domesticated. It is more likely that IT82932 is just one of the highly diverged wild soybean accessions in natural soybean population instead of the direct wild progenitor of cultivated soybeans, which may have been extinct after successive changes towards the transition to cultivated soybeans through domestication.

On the other hand, semi-wild soybean accessions, taxonomically described as a distinct species *Glycine gracilis*, were found in many soybean planting regions in China [15]. In general, these accessions have semi-erect plants with seeds larger than those of *G. soja* [16]. Thus, they were either considered as evolutionary intermediates between *G. soja* and *G. max* [17, 18] or hypothesized as hybrids between them [16, 19]. Recent genotyping-by-sequencing of 72 *G. soja* accessions, 404 *G. max* accessions, and 36 *G. gracilis* accessions suggests that *G. gracilis* is likely to be a transitional species derived from the evolutionary process of domesticated soybean [5, 10], instead of hybrids between *G. soja* and *G. max*. Nevertheless, this study proposed the occurrence of gene flow from wild soybean to cultivated soybean subpopulations [5].

Introgression by hybridization has been recognized as an important process that occurs to some degree between crops and WRC, including both self-pollinated and cross-pollinated species [20–23], as the major avenue for gene flow. For example, despite the existence of pre-zygotic and phenological barriers to hybridization between cultivated maize (*Zea mays*) and its wild progenitor teosinte (*Zea mays* ssp. *mexicana*), introgression between the two taxa were detected in both directions of gene flow [22]. More extensive gene flow was detected between cultivated rice (*Oryza sativa*) and its wild progenitor species (*Oryza rufipogon*) and between two subspecies of the cultivated rice (*O. sativa indica* and *O. sativa japonica*) based on population structure and admixture analyses [21, 23]. Such extensive introgression appears to be responsible for controversial conclusions regarding the domestication history of rice drawn from different studies [24, 25].

Although inter-subpopulation introgression has been revealed by inter-subpopulation admixture in many plant species [5, 22, 23, 25, 26], very few studies have investigated the processes, patterns, and evolutionary consequences of genomic introgression during crop domestication. As *G. max* is able to hybridize with *G. soja* to produce fertile seeds, cross-fertilization between the two taxa would have naturally occurred during soybean domestication. Here, we report the evolutionary patterns and consequences of putative interspecific introgression revealed by comparative analysis of both the nuclear and chloroplast genomes of a diverse panel of *G. soja* and *G. max* (landrace) accessions that are highly representative of the natural genetic diversity of soybean. Our results not only reveal evolutionary factors reshaping the genomic and genetic variation in the wild and cultivated soybean populations but also exemplify the complexity and dynamics of the domestication processes for crops that were even under single domestication.

## Results

### Genome-wide identification of putative *G. soja*-*G. max* introgression

To investigate genome-wide introgression between cultivated and wild soybeans, we analyzed the whole genome resequencing data from a representative soybean population that includes 62 *G. soja* accessions, 130 landraces, and 110 improved soybean cultivars collected from diverse eco-geographic regions in China and from other countries including Korea, Japan, Russia, the USA, and Canada [7]. These samples were distributed in nearly all major phylogenetical clades/groups of 18,480 domesticated soybean accessions and 1168 wild soybean accessions collected from 84 countries or developed in the USA that are deposited in the US Department of Agriculture (USDA) Soybean Germplasm Collection [6] and thus considered to be very representative of soybean genetic diversity. We first identified local regional haplotypes in each of the 62 *G. soja* and 240 *G. max* accessions that were identical by descent (IBD) to individuals within the *G. soja* and *G. max* subpopulations using all SNP data from the 302 accessions following an approach previously described [27]. To calculate the frequencies of the shared haplotypes in different regions along each chromosome of the soybean genome, we divided each chromosome into bins of 10 kb and calculated the numbers of recorded IBD tracts between each accession and the two *G. soja* and *G. max* subpopulations per bin by pairwise comparisons. These numbers were normalized from 0 (no IBD detected) to 1 (IBD shared by all individuals within a subpopulation), and the normalized IBD between each accession and the *G. soja* subpopulation (nIBD_*G. soja*_) and between each accession and the *G. max* subpopulation (nIBD_*G. max*_) were used to calculate the relative IBD (rIBD) between the compared groups (rIBD = nIBD_*G. soja*_ − nIBD_*G. max*_). Finally, the putative genomic introgression from the *G. soja* subpopulation to each of the cultivated soybean accessions and from the *G. max* subpopulation to each of the wild soybean accessions was identified.

**Fig. 1:**
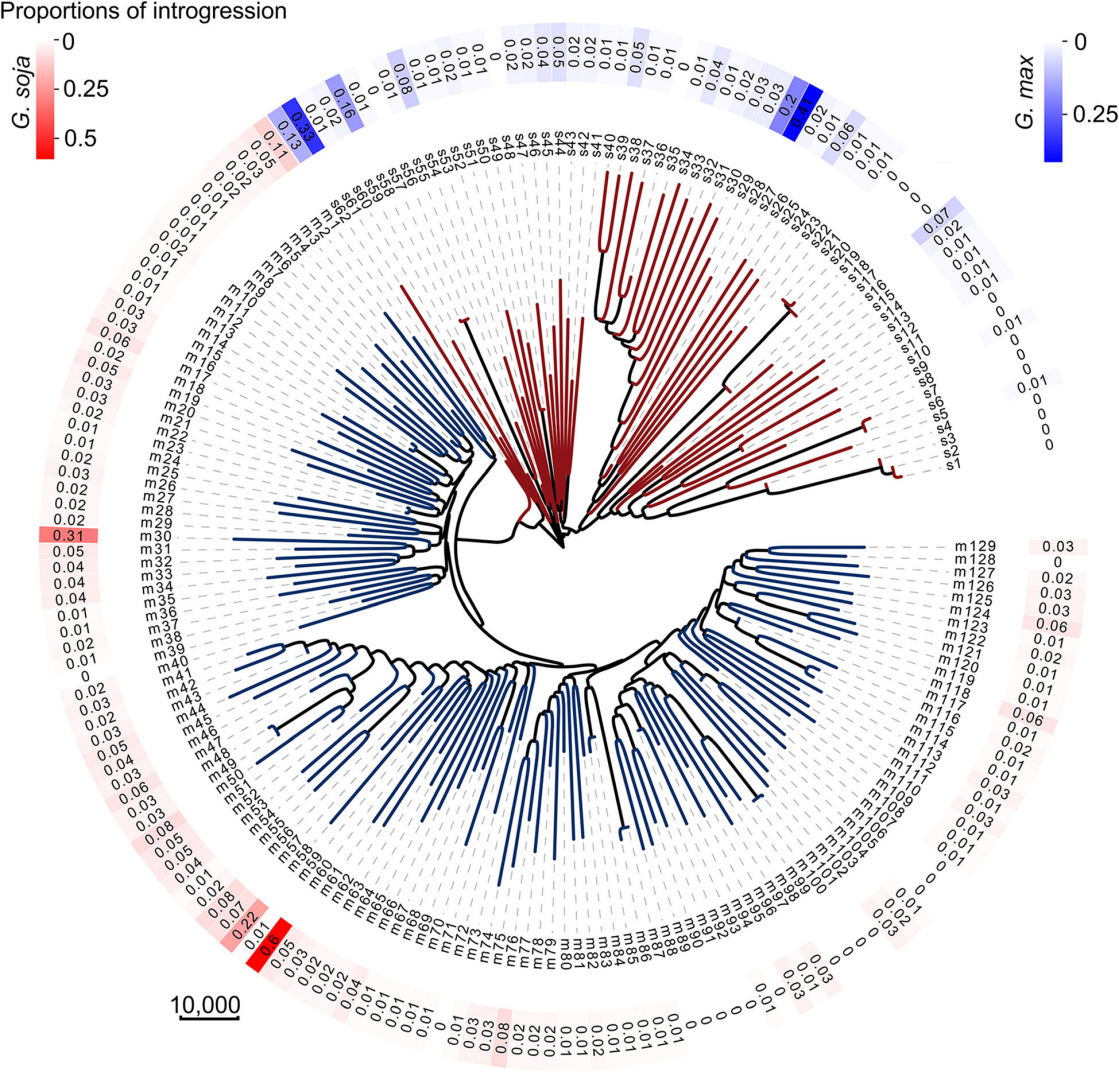
Phylogenetic neighbor-joining tree of a panel of wild and cultivated soybeans. The tree was constructed based on genome-wide SNPs detected by genome resequencing using the MEGA program. Branches labeled in red and blue indicate *G. soja* and *G. max* (landrace), respectively. The codes for individual accessions and the fraction of introgressed fragments are shown in the out layers of the tree

**Fig. 2:**
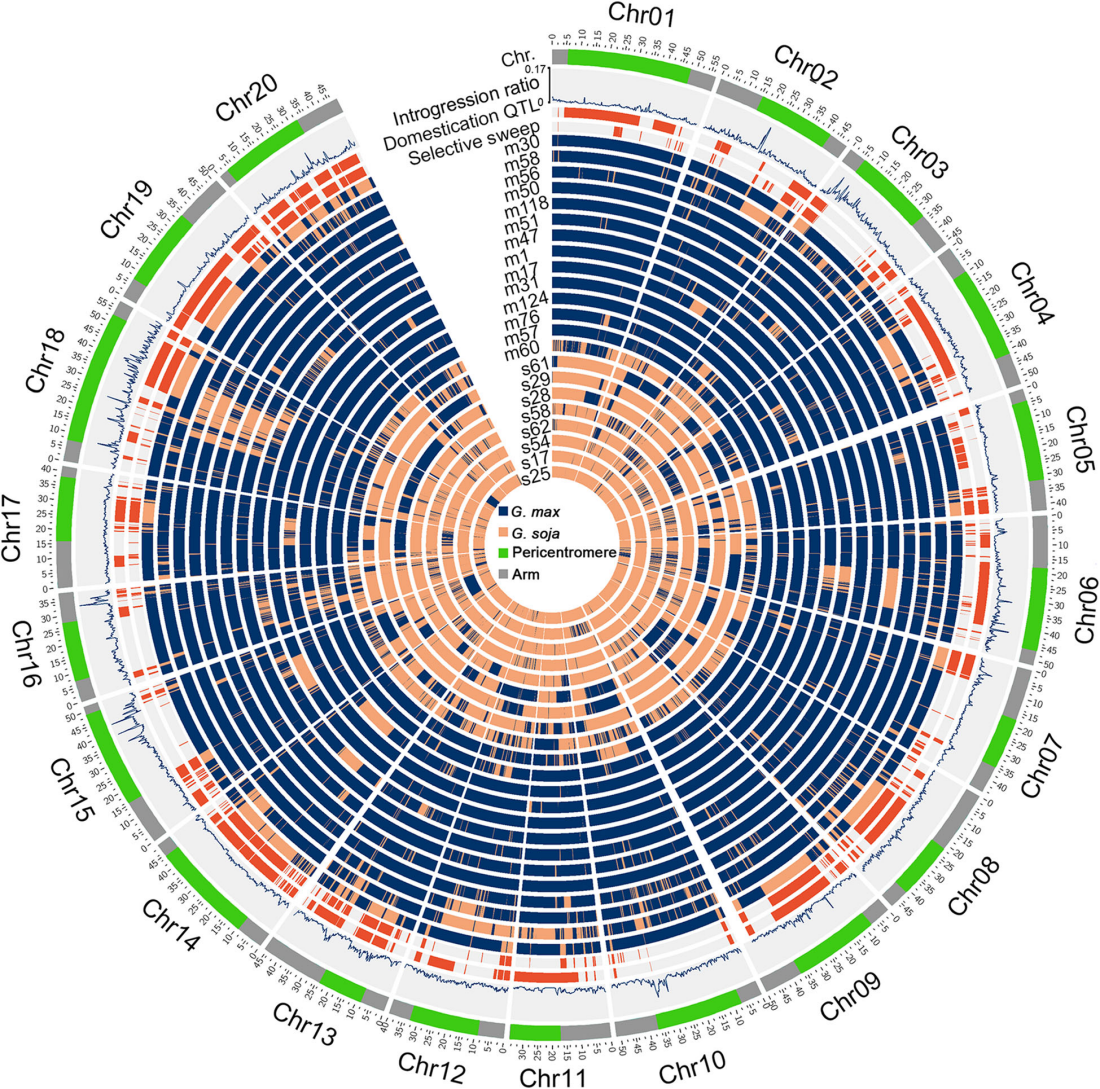
Genome-wide distribution of interspecific introgression and genomic features. The rings of the circle, from outside to inside, show chromosomes including (i) chromosome arms (gray color) and pericentromeric regions (green color), (ii) chromosomal distribution of introgression rates in the whole population, (iii) chromosomal distribution of domestication-related QTL as indicated by red bars in corresponding ring, (iv) chromosomal distribution of selective sweeps as indicated by red bars in corresponding ring, (v) chromosomal distribution of interspecific introgression in each of the 12 *G. max* and 10 *G. soja* accessions, as indicated by the 22 rings, whose genomes each possesses more than 5% introgressed fragments. In these 22 rings, the *G. max* segments were shown in blue and the *G. soja* segments were shown in orange

Through this approach, 297 of the 302 accessions were detected to contain putative interspecific introgression (Additional file 1: Table S1, Additional file 2: Figure S1). The 110 elite varieties were excluded from further analyses because these varieties exhibited relatively smaller rates of introgression from the wild soybean (0.00015~0.03) and because the development of these varieties involved human-made hybridization that could obscure naturally occurring introgression events. One landrace was also excluded from further analyses due to its relatively low quality of genomic sequences and high level of heterozygosity. Among the remaining accessions including 62 *G. soja* accessions and 129 landraces, the proportions of detected introgressed fragments in individual genomes range from 0.00037 to 0.60, with an average of 0.032 (Fig. 1, Additional file 1: Table S1). The chromosomal distribution of the detected introgression in accessions with > 0.05 (5%) introgressed fragments in individual genomes (Additional file 1: Table S1) are illustrated in Fig. 2. Among the *G. soja* genomes, the proportions of detected *G. max* fragments range from 0.00059 to 0.41, with an average of 0.019 (Additional file 2: Figure S1, upper panel). Among the *G. max* genomes, the proportions of detected *G. soja* fragments range from 0.00037 to 0.60, with an average of 0.031 (Additional file 2: Figure S1, lower panel). In total, 43.94% and 54.61% of the putative introgressed fragments in the *G. soja* and *G. max* subpopulations are shared by two or more accessions, and the remaining are accession-specific (Additional file 2: Figure S2). None of the putative introgressed fragments detected in this study were fully fixed in either the *G. soja* or *G. max* subpopulations (Fig. 2).

**Fig. 3:**
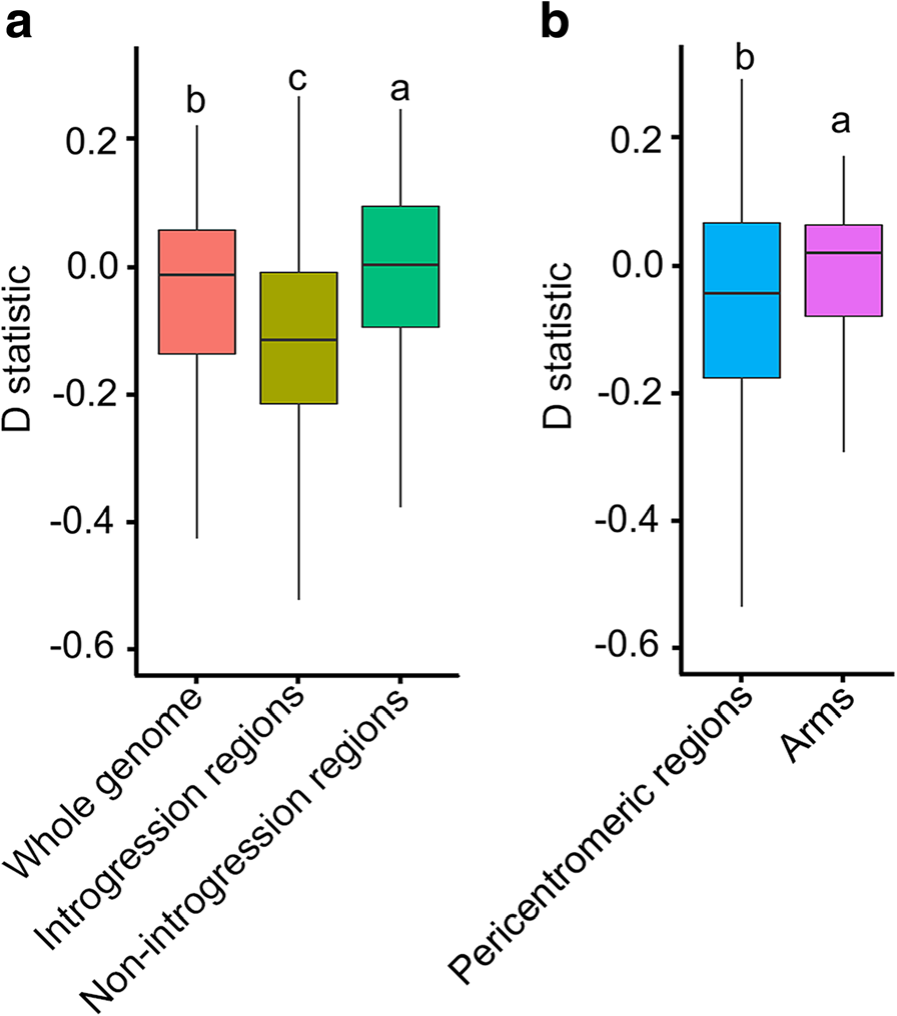
*D*-statistic analysis reveals distinct patterns of gene flow in different regions between *G. soja* and *G. max*. **a** *D*-statistic for large putative *G. max*-introgressed regions detected in the 8 *G. soja* accessions/large putative *G. soja*-introgressed regions detected in the 14 *G. max* accessions in pairwise comparison with those regions in randomly selected 10 *G. max* accessions/10 *G. max* accessions without detected interspecific introgression versus regions without putative introgression. **b** *D*-statistic for pericentromeric regions versus chromosomal arms in the pairwise comparison between accessions as described in **a**. Significant differences (*p* values < 0.05) between compared regions, as indicated by different letters in the boxblots, were detected by the Kolmogorov-Smirnov test

Previous analyses of population structure and admixture in soybeans have revealed local genomic regions showing exceptional similarities between *G. soja* and *G. max* (5, 7), which were deemed as evidence of genomic introgression. The rIBD analysis defining the local genomic regions of individual accessions involved in putative introgression described above provides further evidence in support of *G. soja-G. max* introgression. Nevertheless, there remains a possibility that some of the putative introgression, particularly putative *G. soja* fragments detected in the *G. max* background, could be resulted from incomplete lineage sorting of extant ancestral polymorphisms in the source population used in the domestication process. In an attempt to garner additional evidence to support the hypothesis of introgression, we conducted *D*-statistic analysis for the large putative *G. max*-introgressed regions detected in the 8 *G. soja* accessions (Fig. 2) in pairwise comparison with 10 randomly selected *G. max* accessions without detected introgression (Fig. 1) and for the large putative *G. soja*-introgressed regions detected in the 14 *G. max* accessions (Fig. 2) in pairwise comparison with 10 randomly selected *G. soja* accessions without detected introgression (Fig. 1). As shown in Fig. 3a, the *D*-statistic for the regions harboring putative introgression (*D* = − 0.15 ± 0.07) was significantly lower than the regions without putative introgression (*D* = − 0.08 ± 0.05) and was also significantly lower than the genome-wide average (*D* = − 0.11 ± 0.04), suggesting that gene flow between the *G. soja* and *G. max* taxa was involved in these genomic regions with detected putative introgression.

**Fig. 4:**
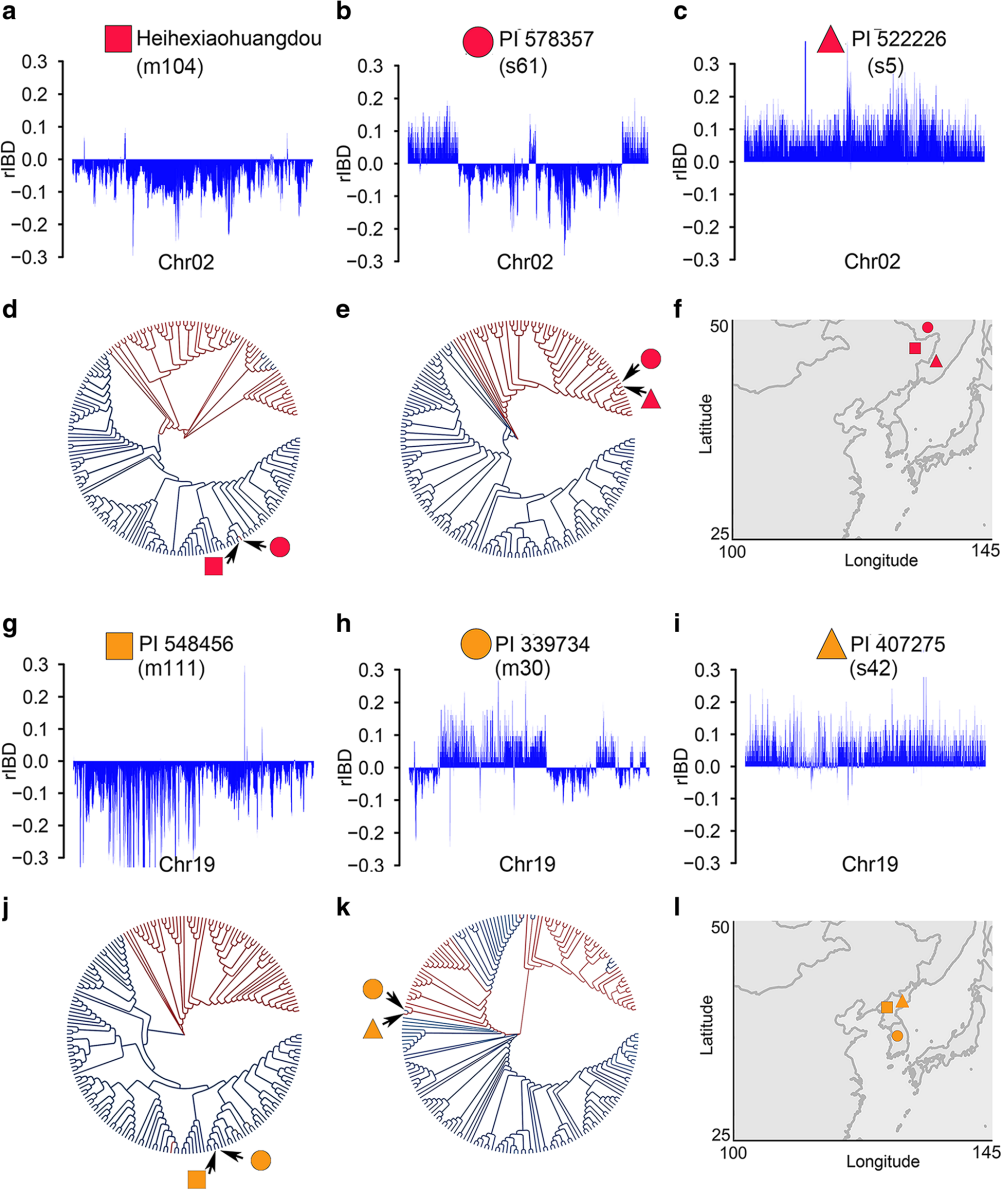
Exemplification of introgression and putative origins of introgression illustrated by relative identical by descent (rIBD). **a**–**c** Genomic component along chromosome 2 in three related accessions Heihexiaohuangdou, PI 578357, and PI 522226. A *G. soja* accession (**b**) carries introgressed *G. max* fragments sharing the highest similarity to the corresponding regions of a landrace (**a**), and the *G. soja* segments sharing highest similarity to the corresponding regions of a *G. soja* accession (**c**). **d** The cladogram tree based on SNPs in the introgression regions of chromosome 2 showing the *G. max* (Heihexiaohuangdou, blue) origin of the regions in *G. soja* accession PI 578357, as pointed by the arrow. **e** The cladogram tree based on SNPs in the non-introgression regions showing the *G. soja* (PI 522226, red) origin of the regions in PI 578357, as pointed by the arrow. **f** Geographic distribution of the three related accessions. **g**–**i** Genomic component along chromosome 19 in three related accessions PI 548456, PI 339734, and PI 407275. A *G. max* accession (**h**) carries introgressed *G. soja* fragments sharing the highest similarity to the corresponding regions of a *G. soja* accession (**i**) and the *G. max* segments sharing the highest similarity to the corresponding regions of a *G. max* accession (**g**). **j** The cladogram tree based on SNPs in the non-introgression regions showing the *G. max* (PI 548456, blue) origin of the regions in PI 339734, as pointed by the arrow. **k** The cladogram tree based on SNPs in the introgression regions of chromosome 2 showing the *G. soja* (PI 407275, red) origin of the regions in *G. max* accession PI 339734, as pointed by the arrow. **l** Geographic distribution of the three related accessions

In an attempt to track the origin of the detected introgression, we compared large and representative introgression segments in a *G. soja* accession (PI 578357, s61) and a landrace (PI 339734, m30), which were estimated to carry 33% and 31% introgressed fragments, respectively, with corresponding regions in other accessions. Genome-wide putative introgression in a *G. soja* accession PI 578357 (s61), one of the *G. soja* accessions adjacent to the *G. max* clades, was exemplified in Fig. 4b and Additional file 2: Figure S3.

The introgression regions in PI 578357 span the entire pericentromeric region of chromosome 2 without previously detected domestication QTLs [28] (Fig. 2) and were thus considered to have undergone minimal selective pressure for their retention. We found that the examined regions of chromosome 2 in PI 578357, grown in Amur, Russia, share the highest sequence similarity with their corresponding regions in a landrace Heihexiaohuangdou (m104) grown in Heihe, China (Fig. 4a, b, d, f). By contrast, the non-introgression regions of PI 578357 share the highest similarity with their corresponding regions in a *G. soja* accession PI 522226 (s5) grown in Primorye, Russia (Fig. 4b, c, e, f), which is one of the *G. soja* accessions that are most diverged from PI 578357 and from the *G. max* lineage (Fig. 1), and thus is unlikely to be involved in or derived from the source population used for soybean domestication. The introgression regions of chromosome 19 in PI 339734, a landrace grown in Korea, were found to share the highest sequence similarity with their corresponding regions in a *G. soja* accession PI 407275 (s42) that was also grown in Korea (Fig. 4h, i, k, l). By contrast, the non-introgression regions of PI 339734 share the highest similarity with their corresponding regions in a landrace PI 548456 (m111) that was also grown in Pyongyang, North Korea (Fig. 4g, h, j, l). The geographic distribution, the patterns and levels of sequence similarity and divergence of local genomic regions, and the whole genome-wide sequence diversity and phylogeny of these accessions together suggest that the detected chimerism of chromosomes, as described above, was most likely resulted from interspecific introgression instead of incomplete lineage sorting of ancestral polymorphisms in the source population for soybean domestication. Based on gene sequences in the entire genome, the divergence times between PI 578357 and Heihexiaohuangdou and between PI 339734 and PI 407275 were dated to ~ 0.37 and 0.27 million years ago (mya), respectively. As soybean domestication occurred only ~ 6000–9000 years ago [3, 29], such a high level of similarities of the examined introgression regions between the two pairs of (*G. soja*-*G. max*) accessions exemplified above should be considered as direct evidence of *G. soja*-*G. max* introgression.

### Factors shaping the landscape of *G. soja*-*G. max* introgression

To understand the evolutionary forces shaping the distribution of genomic introgression in the two subpopulations, we first compared the average proportions of introgressed fragments between pericentromeric regions and chromosomal arms that were roughly defined based on the rates of local genetic recombination and physical positions of centromere-enriched repeats in the soybean genome [12, 30]. In general, pericentromeric regions exhibit severely reduced rates of genetic recombination and biased accumulation for deleterious mutations such as the insertion of transposable elements compared with chromosomal arms [13, 30, 31]. We found that, despite some exceptions, overall the pericentromeric regions have higher proportions of introgressed fragments in either the *G. soja* subpopulation or the *G. max* subpopulation (paired Student *t* test, *p* value < 0.01, Fig. 2 and Additional file 3: Table S2). *D*-statistic analysis for pericentromeric regions in comparison with arms was performed using the same subset of *G. max* and *G. soja* accessions. As shown in Fig. 3b, the *D*-statistic for the pericentromeric regions (*D* = − 0.12 ± 0.06) was significantly lower than the chromosome arms (*D* = − 0.09 ± 0.02) (Fig. 3b), suggesting a biased accumulation of gene flow in pericentromeric regions. Such a bias may be partially the outcome of the reduced rates of genetic recombination and thus reduced efficiency in purging introgressed variation/fragments in pericentromeric regions compared with chromosomal arms.

**Fig. 5:**
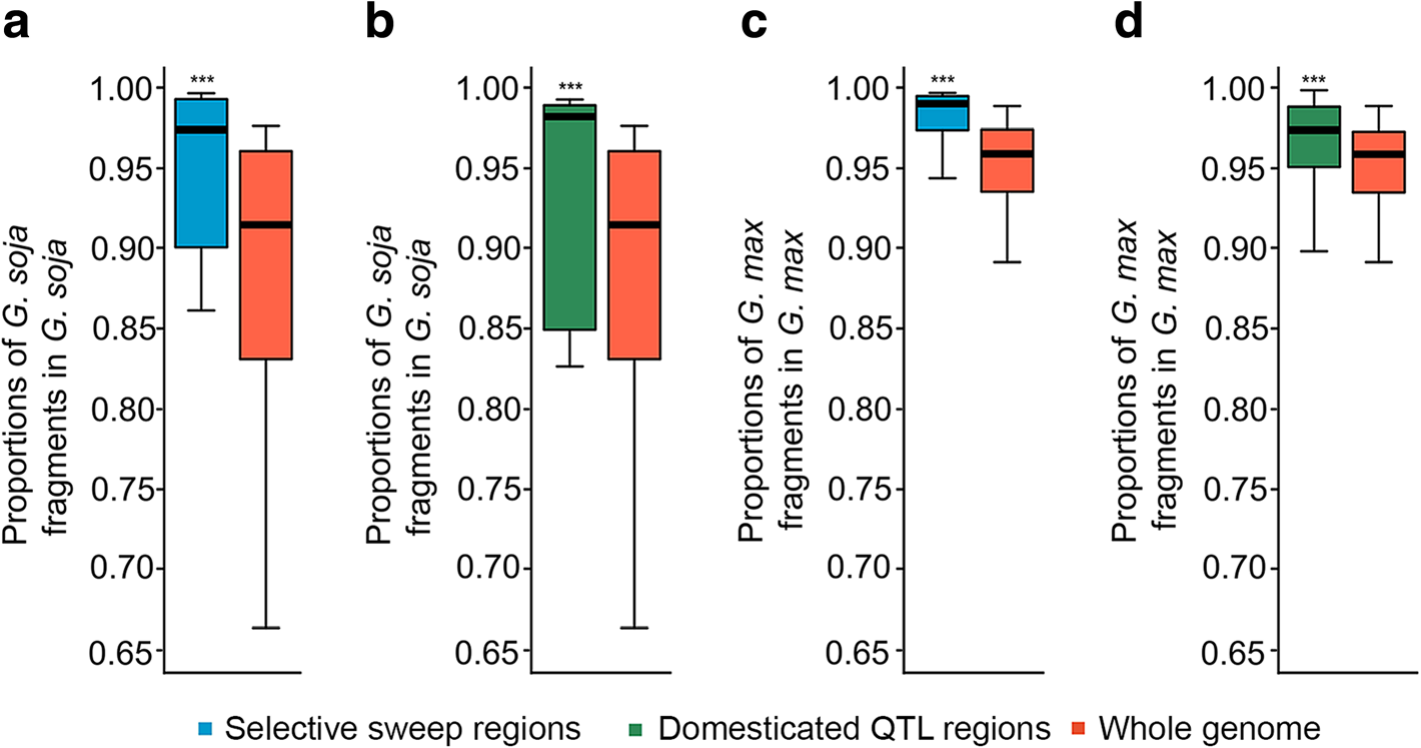
Patterns of natural selection and artificial selection against genomic introgression. **a** Proportions of introgressed *G. max* fragments in selective sweep regions compared with those in the whole genome detected in the *G. soja* subpopulation. **b** Proportions of introgressed *G. max* fragments in domestication-related QTL regions compared with those in the whole genome detected in the *G. soja* subpopulation. **c** Proportions of introgressed *G. soja* fragments in selective sweep regions compared with those in the whole genome detected in the *G. max* subpopulation. **d** Proportions of introgressed *G. soja* fragments in domestication-related QTL regions compared with those in the whole genome detected in the *G. max* subpopulation. Significant difference in each comparison was calculated by paired Student *t* test. ***p < 0.001

Theoretically, genomic introgression resulted from spontaneous hybridization, and subsequent backcrossing involving *G. soja* and *G. max* should have undergone two distinct selection pressures: natural selection for the wild traits towards the formation of *G. soja* or *G. soja*-like accessions adaptive to natural environments versus artificial selection for cultivated traits towards the development of *G. max* or *G. max*-like accessions suitable for cultivation. If this is the case, we would anticipate to observe distinct patterns of distribution of introgressed fragments between the *G. soja* and *G. max* subpopulations. To test this hypothesis, we first estimated the proportions of introgressed fragments in 122 selective sweeps (Fig. 2) that exhibited severe reductions of nucleotide variation from the *G. soja* subpopulation to the *G. max* subpopulation [7]. These regions in cultivated soybeans were likely resulted from strong selective pressure acting on particular loci associated with soybean domestication [8, 9]. As expected, a significantly lower proportion of *G. max* fragments in the regions corresponding to the selective sweep regions in comparison with the remaining part of the genome was detected in the *G. soja* accessions (paired Student *t* test, *p* value = 0.002667, Fig. 5a). By contrast, a significantly lower proportion of *G. soja* fragments in the selective sweep regions in comparison with the remaining part of the genome was detected in the *G. max* accessions (paired Student *t* test, *p* value = 2.542e−09, Fig. 5c).

In addition, we estimated the proportions of introgressed fragments in 44 QTL regions underlying soybean domestication (Fig. 2). These QTLs have been recently identified using approximately 800 recombinant inbred lines (RIL) derived from crosses between Williams 82 and each of 2 *G. soja* accessions PI 468916 (s14) and PI 479752 (s15) [28]. We found that the proportion of introgressed fragments in the corresponding domestication QTL regions of either the *G. max* or *G. soja* accessions is even smaller than detected in the selective sweep regions (Fig. 5b, d). Together, these observations indicate distinct outcomes and effects of bidirectional selection, i.e., natural selection versus artificial selection, on the retention of introgressed fragments in the *G. soja* and *G. max* subpopulations.

### Introgression-mediated gene flow surrounding key domestication genes

Genomic introgression has been realized as a major avenue for gene flow; we wondered how gene flow may have affected the domestication process and the genetic architecture of the soybean genome at a population level. Recently, two key soybean domestication genes *GmHs1-1* and *Bloom1 (B1)* which control seed hardedness and seed coat bloom, respectively, have been isolated [8, 9]. Seed coat impermeability and bloom were considered to be important or essential for the long-term survival of wild soybeans, whereas permeable seed coat without bloom was desirable for agricultural production and human consumption and targeted for selection under domestication. The causal mutation at each of the two loci for the key domestication transition was identified and functionally validated [8, 9]. A recessive mutation (C→T) in the coding region of *GmHs1-1* resulted in the transition from impermeable seed coat in wild soybean to permeable seed coat in cultivated soybean, and selection for the domesticated allele *Gmhs1-1* formed an ~ 160-kb selective sweep region [8]. A recessive mutation (C→T) in the coding region of *B1* was responsible for the loss of seed coat bloom in the cultivated soybeans, and selection of the domesticated allele *b1* resulted in a ~ 301-kb selective sweep region [9]. To our knowledge, these are only two genes identified to date whose two alleles can nearly exclusively distinguish the wild soybeans from the cultivated soybeans, and meanwhile, the identical causal mutation at each of the two loci for the domesticated phenotypes is shared by the cultivated soybeans.

**Fig. 6:**
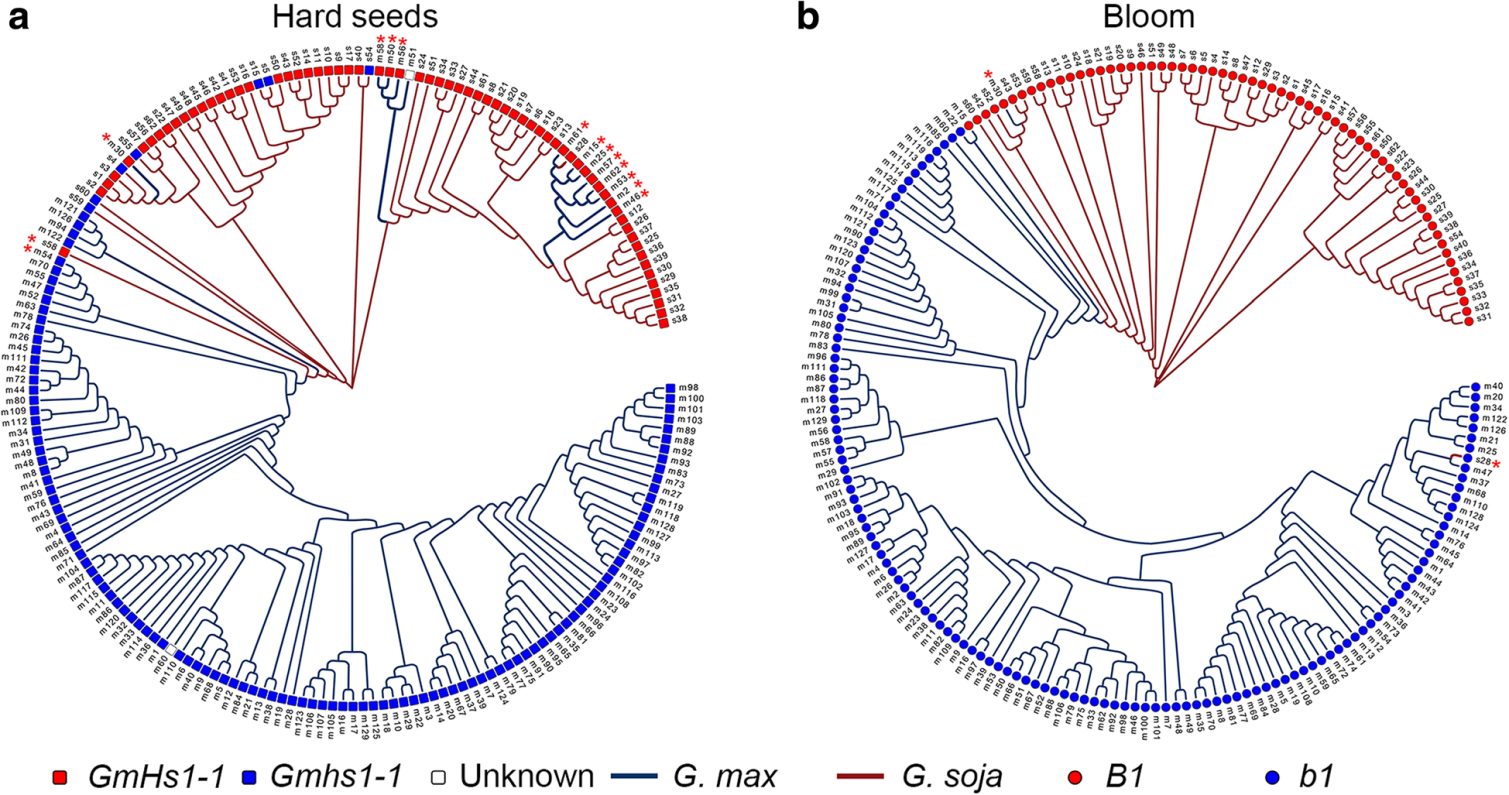
Phylogenetic relationships among the 191 accessions revealed by comparison with two selective sweep regions. **a** The *GmHs1-1* region and **b** the *B1* region. Asterisks mark the *G. soja* and *G. max* accessions containing introgressed fragments from *G. max* and *G. soja*, respectively

Using SNPs in the two selective sweep regions surrounding the *GmHS1-1*/*Gmhs1-1* and *B1*/*b1* loci, we constructed the phylogenetic relationships among the 62 *G. soja* accessions and 129 landraces. Putative introgression involving the 2 selective sweep regions was defined by the phylogenetic relationships. In the ~ 160-kb *GmHs1-1*/*Gmhs1-1* region, 13 landraces were revealed to possess *G. soja*-like *GmHs1-1* region, while only 1 *G. soja* accession, PI 366121 (s58), was found to have the *G. max*-like *Gmhs1-1* region (Fig. 6a). The *G. soja*-like *GmHs1-1* regions found in the 13 landraces were scattered in all the major clades of the *G. soja* population (Fig. 6a), while only a subset of the clades of the *GmHs1-1* regions may be the source for selection for the *Gmhs1-1* allele during domestication. Thus, the diverse *GmHs1-1* regions in these landraces were more likely resulted from interspecific introgression. In the ~ 301-kb *B1/b1* region, PI 339734 was revealed to be the only landrace possessing the *G. soja*-like *B1* region, while PI 549046 (s28) was found to be the only *G. soja* accession containing the *G. max*-like *b1* region (Fig. 6b), which is nearly identical to that of a *G. max* variety PI 437654 (m47) (Fig. 6b). This *G. soja* accession was phylogenetically grouped, at the whole genome level, into a clade that is distant from other *G. soja* clades adjacent to *G. max* (Fig. 1), suggesting that the *b1* region in this *G. soja* accession was likely to rise from *G. max* through gene flow. Among these landraces, PI 339734 is the only accession processing both the *GmHs1-1* and *B1* regions (Fig. 6a, b).

Selective sweeps surrounding domestication QTL are generally detectable at population levels, but the boundaries of specific haplotypes under selection within a particular selective sweep region vary among individual accessions. To further track the footprints of gene flow involving these key domestication loci, we zoomed in at the two domestication genes and their adjacent sequences including respective presumable promoter and terminator sequences, with a focus on the causative mutation that resulted in the domestication allele at each locus. At the *GmHs1-1/Gmhs1-1* locus, 13 landraces were found to share the *G. soja* or *G. soja*-like haplotypes including the *GmHs1-1*-specific nucleotide (C), while 7 *G. soja* accessions were found to possess the *G. max* or *G. max*-like haplotype including *Gmhs1-1*-specific nucleotide (T) (Fig. 6a and Additional file 2: Figure S4). Consistent with the phylogeny of the *B1/b1* sweep regions among the 191 accessions (Fig. 6b), *G. soja* PI 549046 (s28) was found to possess the *b1* haplotype, including the causal mutation (T) for *b1*, which is identical to that of 191 landraces, while the haplotype of landrace PI 339734 (m30) is highly identical to those of the majority of *G. soja* accessions including *B1*-specific nucleotide (C) (Additional file 2: Figure S5). Given the phylogenetic distinction of the *G. soja* and *G. max* subpopulations, the detected admixture of the selective sweep regions and haplotypes at the two domestication gene loci in the investigated population would be considered as further evidence of inter-subpopulation gene flow.

### Introgression revealed by asymmetric diversification between the nuclear and organellar genomes

**Fig. 7:**
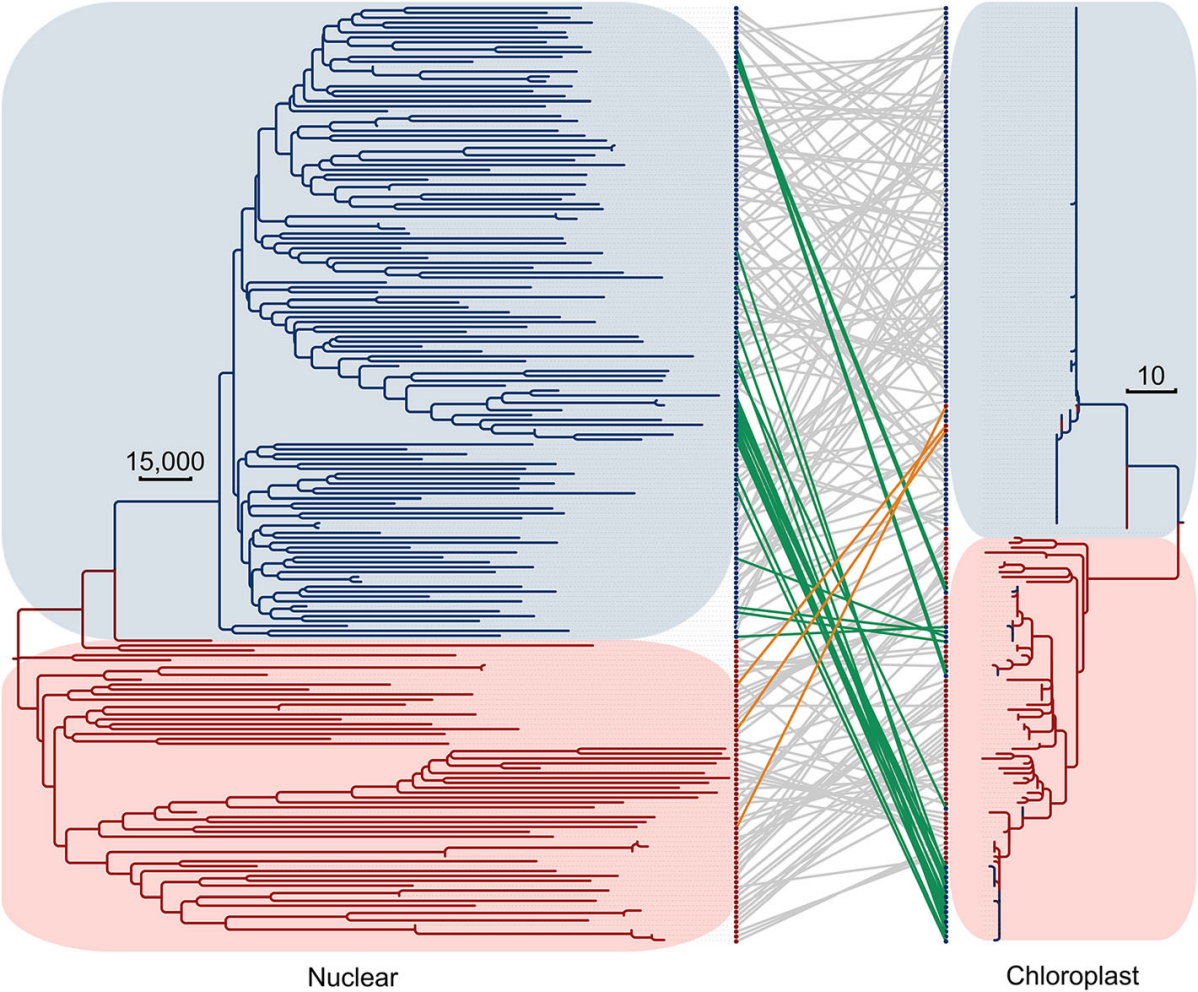
Asymmetric divergence of the nuclear and chloroplast genomes between *G. max* and *G. soja* accessions. The phylogenetic tree of the nuclear genomes of the 191 accessions was constructed using all SNPs detected in the whole genome, while the phylogenetic tree of the chloroplast of the same set of accessions was constructed using 333 highly confident SNPs distributed across the chloroplast genome. The *G. max* accessions were indicated by blue branches of 2 trees and marked by blue dots while the *G. soja* accessions were indicated by red branches of 2 trees and marked by red dots. The same accessions in the 2 trees were connected by lines. Blue lines indicate *G. max* accessions possessing the *G. soja*-type chloroplasts, orange lines indicate *G. soja* accessions possessing the *G. max*-type chloroplasts, and gray lines indicate *G. soja* and *G. max* accessions possessing *G. soja*- and *G. max*-types of chloroplasts, respectively

Given that crosses between *G. soja* and *G. max* can be readily made to produce fertile seeds, the detected introgression or gene flow in this study as described above was thought to be relics of rounds of spontaneous hybridization involving these two gene pools through pollen dissemination and/or seed dispersal. In theory, some of the hybridization events would be detected by comparison with their organellar genomes in the context of the genetic architecture defined by their nuclear genomes. We thus analyzed the chloroplast genome sequences of the 191 re-sequenced soybean accessions [7, 32]. Among the 191 chloroplast genomes, a total of 333 highly accurate SNPs were identified and then used to construct the phylogenetic tree (Fig. 7). Overall, the 191 chloroplast genomes were clustered into 2 subgroups, the *G. max* subgroup and the *G. soja* subgroup. As expected, the chloroplast genomes of the *G. max* accessions within the *G. max* subgroup are less diverged than those of the *G. soja* accessions within the *G. soja* subgroup. Despite the clear distinction of the chloroplast genomes between the *G. max* and *G. soja* subgroups, 24 *G. max* accessions were clustered into the *G. soja* subgroup and 3 *G. soja* accessions were clustered into the *G. max* subgroup (Fig. 7). These observations indicate the occurrence of *G. soja*-*G. max* hybridization events with either *G. soja* or *G. max* as the maternal parent. Such events have apparently reshaped the genetic architecture of the nuclear genomes of both the *G. soja* and *G. max* subpopulations.

For the *G. max* and *G. soja* accessions possessing respective *G. max* and *G. soja* chloroplasts, more closely related accessions according to their nuclear genomes tend to share identical or more similar haplotypes of their chloroplast genomes (Fig. 7 and Additional file 2: Figure S6), suggesting co-evolution between the nuclear genomes and the chloroplast genomes as a general pattern. However, apparent exceptions were also observed. For example, some accessions with highly diverged nuclear genomes were detected to share identical or nearly identical haplotypes of the chloroplast genomes, and some accessions with more closely related nuclear genomes were detected to have more diverged haplotypes of the chloroplast genomes (Additional file 2: Figure S6). Such exceptions suggest that hybridization events between highly diverged accessions in terms of their nuclear genomes within the *G. max* subpopulation or within the *G. soja* subpopulation have also occurred, and such events are very likely to be responsible for the observed unparalleled varietal diversification between the nuclear and chloroplast genomes.

## Discussion

### The consequences of selection against introgression

Wild soybeans are widely distributed over a wide range of eco-geographical regions of East Asia including China, the Russian Far East, the Korean Peninsula, and Japan [33]. To survive under such diverse climate and environment, wild soybeans were diverged for a set of adaptation traits such as flowering periods and maturity that are extremely important for the plant’s photoperiod response [34]. On the other hand, the majority of wild soybean accessions share a set of similar characteristics including a procumbent or climbing growth habit and prolific branching, with dehiscent pods and small black seeds with impermeable seed coat and a layer of seed coat bloom [17]. Apparently, these characteristics were advantageous for the long-term survival of wild soybeans, and thus, genes/QTLs underlying these characteristics in wild soybeans were under strong purifying selection over evolutionary time [8, 9, 35]. Compared with the wild progenitor species, cultivated soybeans show upright growth dominated by a single stem, with non-shattering pods and large shiny seeds covered by with yellow and permeable seed coat, a set of domestication-related trait (DRT) that were favorably selected by ancient farmers for production of soybean seeds as a source of food. Of course, the maintenance of these DRT “adapted” for cultivation reply on sustained agricultural practices.

**Fig. 8:**
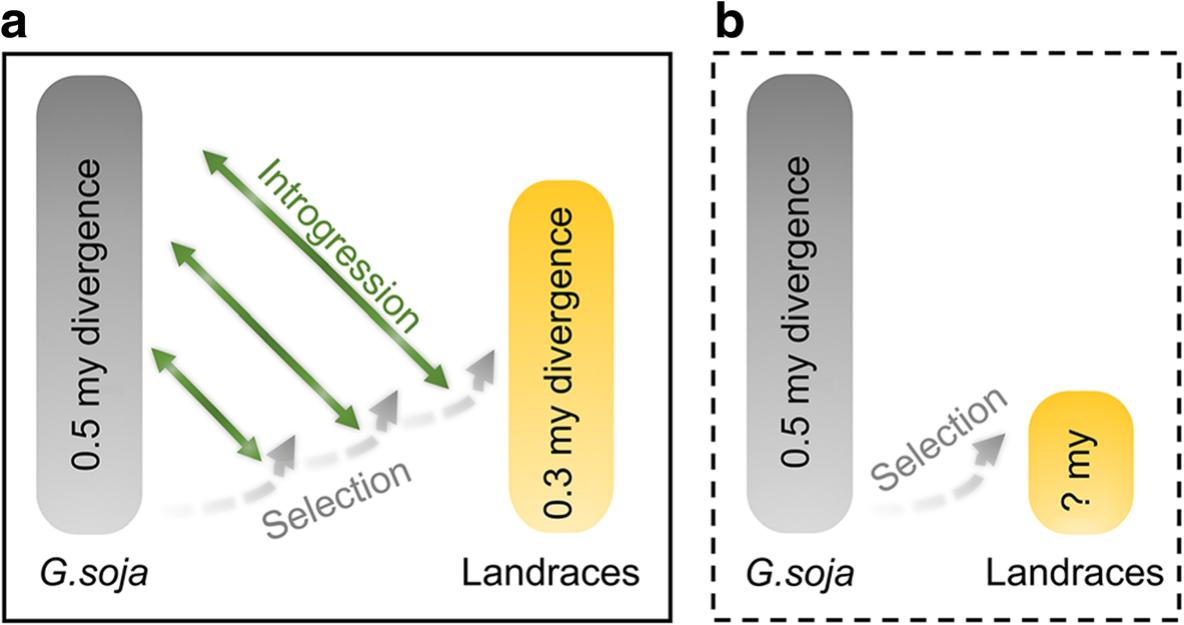
Models of the domestication process of soybean. **a** Rounds of hybridization involving diverse *G. soja* accessions counteracted genetic bottleneck during domestication and contributed to rapid varietal diversification and adaptation of landraces to diverse environments, a model supported by this study. Lines with arrows at both ends indicate rounds of hybridization events involving diverse *G. soja* accessions. The curved arrows indicate recurrent selection against introgression. **b** A hypothetical model of the domestication process of soybean without interspecific introgression that would have resulted in stronger domestication bottleneck

A line of observations gained in this study suggests extensive genomic introgression derived from interspecific hybridization and subsequent backcrossing during or after soybean domestication. Apparently, such events should have been followed by re-selection or recurrent selection for DRT through purging introgressed *G. soja* variation from the QTL or selective sweep regions underlying soybean domestication (Fig. 8). As a result, significantly lower proportions of introgressed *G. soja* fragments were observed in these regions than in the remaining genomic regions of landraces. This pattern echoes the distribution pattern of the putative introgressed *G. max* fragments in the *G. soja* accessions, suggesting that evolutionary fates of interspecific introgression are determined by relative intensities of the two distinct selection pressures.

### The role of introgression in varietal diversification

Despite nearly complete removal of wild-type alleles of domestication-related genes/QTL from landraces, our results indicate that interspecific introgression has played an important role in enhancing the genetic diversity in cultivated soybeans as illustrated in Fig. 8. This proposition was based on the observation that many of the introgressed fragments, as exemplified in Fig. 4e, were from *G. soja* accessions highly diverged from the *G. max* accessions. Given that some of these *G. soja* accessions are most distant from the cultivated soybeans and thus unlikely to be the direct wild progenitor of cultivated soybeans, the introgression involving those *G. soja* accessions would have boosted the genetic diversity of cultivated soybeans to a level that could not be reached through varietal diversification that does not involve interspecific introgression (Fig. 8). Although overall the level of genetic diversity in cultivated soybean subpopulation has been substantially reduced compared with the *G. soja* subpopulation, pairwise comparison estimated the time of divergence among some landraces to > 0.3 mya, very close to the estimated average time (~ 0.37 mya) of divergence among the *G. soja* accessions (Additional file 2: Figure S7), further suggesting potential contribution of *G. soja* to varietal diversification of cultivated soybeans through interspecific introgression. As observed in maize [22], such inter-subpopulation introgression may have contributed to adaptation traits important for radiation of cultivated soybeans to diverse environments beyond the location for soybean domestication.

### Introgression and asymmetric nuclear and organellar genome evolution

Actually, the asymmetric patterns of divergence and phylogenetic relationships between nuclear genomes and chloroplast genomes of the *G. soja* and *G. max* accessions (Additional file 2: Figure S7) not only reveal interspecific introgression between the two taxa but also suggest the occurrence of introgression within either the *G. soja* or *G. max* taxon (Additional file 2: Figure S6). It is obvious that the observed 23 *G. max* accessions with the *G soja*-type chloroplast genome and the 3 *G. soja* accessions processing the *G. max*-type chloroplast genome are indicative of introgression events involving *G. soja* and *G. max*. However, whether an introgression event involving the two taxa possesses heterologous nuclear and chloroplast genomes would be determined by the maternal parent involved in the final hybridization event. Therefore, interspecific introgression events that actually occurred and shaped the genetic architecture of the wild and domesticated soybeans analyzed in this study would outnumber those reflected by the 26 accessions described above.

In addition, due to the limitation of IBD analysis and high levels of sequence similarity, intraspecific introgression events may not be easily detected. In general, the phylogenetic relationships among wild soybean accessions are highly consistent with their geographical distribution [6, 35], but exceptions were also observed, particularly, for the accessions distributed in different eco-regions of China [33, 36, 37]. If asymmetric evolution of the nuclear and chloroplast genomes among cultivated soybeans are indeed indicative of intraspecific hybridization, we envision that such events would have further shaped the genetic architecture of the soybean population, resulting in rapid diversification of local genomic regions among accessions.

## Conclusions

Elucidating evolutionary patterns and consequences of introgression between crops and their WRC is important for understanding the processes and history of crop domestication. By profiling the genome-wide distribution of putative *G. max*-*G. soja* introgression in the context of genomic features, soybean domestication QTL, selective sweeps, and phylogenetic relationships of a panel of representative soybean accessions constructed based on their nuclear and chloroplast genomes, we illustrated the nature of selection against genomic introgression derived from *G. max*-*G. soja* hybridization and subsequent backcrossing, evolutionary forces shaping the genetic and genomic architecture of the *G. max* and *G. soja* population, and potential effects of introgression on rapid varietal diversification for adaption to various climate environments for cultivation. These results provide novel insights into the history and dynamic process of soybean domestication and explain the controversial conclusions/debates in terms of the date and location of soybean domestication from previous studies. Because of such extensiveness of deduced introgression in the soybean population, it is important to analyze and compare local genomic regions underlying domestication-related traits or other traits of interest towards effective utilization of genetic diversity for crop improvement.

## Methods

### Acquisition of the nuclear and chloroplast genome sequences

Nuclear and chloroplast genome sequences of soybean accessions investigated in this study were reported previously [7]. The identities of the accessions, the fractions of introgressed fragments detected in each accession, and the alleles at the *GmHs1-1* and *B1* loci were listed in Additional file 1: Table S1.

### Mapping of short sequence reads and detection of genetic variation

The paired-end resequencing reads were downloaded from SRP045129 in NCBI Short Reads Archive by fastq-dump program by SRA-toolkit (version 2.3.5, https://github.com/ncbi/sratoolkit). Then, the low-quality reads were filtered (Phred quality value < 20) using fastx-toolkit [38] (version 0.0.14, http://hannonlab.cshl.edu/fastx_toolkit/). All cleaned reads were mapped to the soybean reference genome version 1.1 (http://genome.jgi.doe.gov/Phytozome/download/) by BWA program [39] (version 1.7.3) with four mismatches allowed per read. Only uniquely mapped paired reads were used for the detection of genetic variation. The entire selected bam files were converted into BAM, and potential PCR duplicates were removed using the Samtools program [40] (version 1.3.1). Variation was called through the best practice pipeline of the Genome Analysis Toolkit (GATK, version 3.5.0) [41] and Picard-tools programs (picard.sourceforge.net, version 2.0.1) [42]. The SNP VCF files were merged together by VCFtools [43] (version 0.1.14). SNPs with allele frequencies lower than 1% and distances to adjacent SNPs less than 3 bp were excluded in further analyses.

To investigate genetic variation among chloroplast genomes of the same set of soybean accessions, we mapped all chloroplast reads from each accession to the soybean chloroplast genome sequence from Williams 82 (DQ317523.1, Gene bank in NCBI), following the same protocol as used for the nuclear genomes, and a merged SNP matrix of chloroplast genomes was obtained for analysis of genetic variation.

### Pairwise IBD detection

Local regional haplotypes in each *G. soja* or *G. max* accession that were identical by descent (IBD) to individuals within the *G. soja* and *G. max* subpopulations were identified following an approach previously described [27], with minor modification. The approach involves two main steps, IBD region identification and relative IBD (rIBD) statistics. The matrix of genome-wide SNPs from the investigated *G. soja* and *G. max* populations was served as input for the IBD identification pipeline. All the *G. soja* and *G. max* accessions were converted to the beagle format by the in-house Perl scripts and then phased with the fastPhase function of the Beagle program [44] (version 3.3.2). The shared haplotypes between any two of the *G. soja* and *G. max* accessions were detected and extracted with the Beagle fastIBD function of the Beagle program. Phasing and IBD detection were run five times with different thresholds (10^−5^ to 10^−6^), independently, for assigning all possible IBDs to the haplotypes of two accessions. The identified IBD tracks from all the five runs were merged and then extracted using custom Perl scripts.

To profile the frequency of shared haplotypes along individual chromosomes, each chromosome was divided into bins of 10 kb with a sliding window of 1 kb, and the number of recorded IBD tracts between each accession and the two groups (i.e., *G. soja* and *G. max*) of accessions was computed per bin. As the total number of pairwise comparisons differed between the groups, these numbers were normalized from 0 (no IBD detected) to 1 (IBD shared by all individuals within the group) according to the total number of accessions in the group. The normalized IBD between the accession and the *G. soja* group (nIBD_*G. soja*_) and the normalized IBD between this accession and the *G. max* group (relative nIBD_*G. max*_) were then used to calculate the relative IBD (rIBD = nIBD_*G. soja*_ − nIBD_*G. max*_). We profiled rIBD blocks along chromosomes in the order of the 10-kb bins to define putative genomic introgression. The distribution of putative introgression in each of the examined accessions was illustrated using the Circos program [45] (version 0.69).

### *D*-statistic analyses

*D*-statistic has been used to distinguish between the hypotheses of introgression and shared ancestral variation at specific loci [27, 46, 47]. For a specific category of genomic regions, we computed *D*-statistic values for the regions by pairwise comparison between two accessions from two distinct groups, such as the group of *G. soja* accessions with putative *G. max* introgression in the regions versus the group of *G. max* accessions without any putative genomic introgression and the group of *G. max* accessions with putative *G. soja* introgression in the regions versus the group of *G. soja* accessions without any putative genomic introgression. *D*-statistic values were calculated following the protocol previously described [46].

### Estimation and profiling of divergence time

To estimate the divergence time between each two accessions, coding sequences (CDS) from genes that covers ≥ 80% in *G. soja* and *G. max* accessions were used. Rates of synonymous substitution (*Ks*) were calculated using the maximum likelihood (ML) method of the CODEML subprogram in the PAML package [48] (version 4.8, http://abacus.gene.ucl.ac.uk/software/paml.html). The values of *Ks* were converted to divergence time by employing an average substitution rate of 6.1 × 10^−9^ [11].

### Phylogenetic analyses

SNPs from the nuclear genome, chloroplast genome, selective sweep regions (*B1* and *GmHs1-1*) and introgressed regions with MAF > 0.05 and heterozygous rate < 0.50 were used to construct phylogenetic trees using the Neighbor-Join method in MEGA7 [49] and visualized using Evolview [50], an online visualization tool for phylogenetic trees (version 2).

### Statistics analyses

All statistical tests in this paper were performed using basic packages in R language [51] (version 3.3.1).

## Additional files

Additional file 1: **Table S1.** Identities, codes, introgression rates, and genotypes of the accessions used in this study (XLSX 19 KB).

Additional file 2: **Figure S1.** Proportions of introgressed fragments in each of the *G. soja* and *G. max* accessions investigated. **Figure S2.** The statistics of introgression frequency in each window among 22 selected accessions. **Figure S3.** Genome-wide distribution of introgressed *G. max* fragments in a *G. soja* accession PI 578357. **Figure S4.** Haplotypes surrounding the *GmHs1-1* region. **Figure S5. **Haplotypes surrounding the *B1* region. **Figure S6.** Asymmetric divergence of the nuclear and chloroplast genomes within the *G. soja* or *G. max* subpopulation. **Figure S7. **Distribution of divergence time between any two accessions (DOCX 17 MB).

Additional file 3: **Table S2.** Proportions of introgressed fragments in chromosomal arms and pericentromeric regions in the examined soybean population. (XLSX 12 KB). (XLSX 11 kb)

## Abbreviations

CDS: Coding sequence
DRT: Domestication-related trait
IBD: Identical by descent
nIBD: Normalized identical by descent
QTL: Quantitative trait locus
rIBD: Relative identical by descent
RIL: Recombinant inbred line
SNP: Single nucleotide polymorphism
WRC: Wild relative of crop

## References

[1] Hartman GL, West ED, Herman TK (2011). Crops that feed the World 2. Soybean—worldwide production, use, and constraints caused by pathogens and pests. Food Security.

[2] Carter T, Hymowitz T, Nelson R. Biogeography, local adaptation, Vavilov, and genetic diversity in soybean. In Biological resources and migration. Berlin: Springer; 2004. p. 47–59.

[3] Kim MY, Van K, Kang YJ, Kim KH, Lee S-H (2012). Tracing soybean domestication history: from nucleotide to genome. Breed Sci.

[4] Hammer K (1984). Das Domestikationssyndrom. Genet Resour Crop Evol.

[5] Han Y, Zhao X, Liu D, Li Y, Lightfoot DA, Yang Z, Zhao L, Zhou G, Wang Z, Huang L (2016). Domestication footprints anchor genomic regions of agronomic importance in soybeans. New Phytol.

[6] Song Q, Hyten DL, Jia G, Quigley CV, Fickus EW, Nelson RL, Cregan PB (2015). Fingerprinting soybean germplasm and its utility in genomic research. G3 (Bethesda).

[7] Zhou Z, Jiang Y, Wang Z, Gou Z, Lyu J, Li W, Yu Y, Shu L, Zhao Y, Ma Y (2015). Resequencing 302 wild and cultivated accessions identifies genes related to domestication and improvement in soybean. Nat Biotechnol.

[8] Sun L, Miao Z, Cai C, Zhang D, Zhao M, Wu Y, Zhang X, Swarm SA, Zhou L, Zhang ZJ (2015). *GmHs1-1*, encoding a calcineurin-like protein, controls hard-seededness in soybean. Nat Genet.

[9] Zhang D, Sun L, Li S, Wang W, Ding Y, Swarm SA, Li L, Wang X, Tang X, Zhang Z (2018). Elevation of soybean seed oil content through selection for seed coat shininess. Nat Plants.

[10] Sedivy EJ, Wu F, Hanzawa Y (2017). Soybean domestication: the origin, genetic architecture and molecular bases. New Phytol.

[11] Kim MY, Lee S, Van K, Kim T-H, Jeong S-C, Choi I-Y, Kim D-S, Lee Y-S, Park D, Ma J (2010). Whole-genome sequencing and intensive analysis of the undomesticated soybean (*Glycine soja* Sieb. and Zucc.) genome. Proc Natl Acad Sci U S A.

[12] Schmutz J, Cannon SB, Schlueter J, Ma J, Mitros T, Nelson W, Hyten DL, Song Q, Thelen JJ, Cheng J (2010). Genome sequence of the palaeopolyploid soybean. Nature.

[13] Tian Z, Rizzon C, Du J, Zhu L, Bennetzen JL, Jackson SA, Gaut BS, Ma J (2009). Do genetic recombination and gene density shape the pattern of DNA elimination in rice long terminal repeat retrotransposons?. Genome Res.

[14] Beissinger TM, Wang L, Crosby K, Durvasula A, Hufford MB, Ross-Ibarra J (2016). Recent demography drives changes in linked selection across the maize genome. Nat Plants..

[15] Wang K-J, Li X-H, Liu Y (2011). Fine-scale phylogenetic structure and major events in the history of the current wild soybean (*Glycine soja*) and taxonomic assignment of semi-wild type (*Glycine gracilis* Skvortz.) within the Chinese subgenus Soja. J Hered.

[16] Hymowitz T (1970). On the domestication of the soybean. Econ Bot.

[17] Broich SL, Palmer RG (1980). A cluster analysis of wild and domesticated soybean phenotypes. Euphytica.

[18] Broich SL, Palmer RG (1981). Evolutionary studies of the soybean: the frequency and distribution of alleles among collections of *Glycine max* and *G. soja* of various origin. Euphytica.

[19] Fukuda Y (1933). Cytogenetical studies on the wild and cultivated Manchurian soybeans (*Glycine* L.). JPN J BOT.

[20] Mallet J (2005). Hybridization as an invasion of the genome. Trends Ecol Evol.

[21] Zhao K, Wright M, Kimball J, Eizenga G, McClung A, Kovach M, Tyagi W, Ali ML, Tung C-W, Reynolds A (2010). Genomic diversity and introgression in *O sativa* reveal the impact of domestication and breeding on the rice genome. PloS one.

[22] Hufford MB, Lubinksy P, Pyhäjärvi T, Devengenzo MT, Ellstrand NC, Ross-Ibarra J (2013). The genomic signature of crop-wild introgression in maize. PLoS Genet.

[23] Wang H, Vieira FG, Crawford JE, Chu C, Nielsen R (2017). Asian wild rice is a hybrid swarm with extensive gene flow and feralization from domesticated rice. Genome Res.

[24] Sang T, Ge S (2007). The puzzle of rice domestication. J Integr Plant Biol.

[25] Huang X, Kurata N, Wang Z-X, Wang A, Zhao Q, Zhao Y, Liu K, Lu H, Li W, Guo Y (2012). A map of rice genome variation reveals the origin of cultivated rice. Nature.

[26] Zhou Y, Zhang L, Liu J, Wu G, Savolainen O (2014). Climatic adaptation and ecological divergence between two closely related pine species in Southeast China. Mol Ecol.

[27] Bosse M, Megens H-J, Frantz LA, Madsen O, Larson G, Paudel Y, Duijvesteijn N, Harlizius B, Hagemeijer Y, Crooijmans RP (2014). Genomic analysis reveals selection for Asian genes in European pigs following human-mediated introgression. Nat Commun.

[28] Swarm SA, Sun L, Wang X, Wang W, Brown PJ, Ma J, Nelson RL. Genetic dissection of domestication-related traits in soybean through genotyping-by-sequencing of two interspecific mapping populations. Theor Appl Genet. 2019. 10.1007/s00122-018-3272-6 [Epub ahead of print] PubMed PMID: 30607438. 10.1007/s00122-018-3272-6 30607438

[29] Carter TE, Nelson RL, Sneller CH, Cui Z. Genetic diversity in soybean. In soybeans: Improvement, production, and uses. Volume agronomy. Edited by Boerma HR SJ. Am Soc of Agronomy, Crop Sci Soc of Am, Soil Sci Soc of Am, Madison, 2004. p. 303–416.

[30] Du J, Grant D, Tian Z, Nelson RT, Zhu L, Shoemaker RC, Ma J (2010). SoyTEdb: a comprehensive database of transposable elements in the soybean genome. BMC Genomics.

[31] Tian Z, Zhao M, She M, Du J, Cannon SB, Liu X, Xu X, Qi X, Li M-W, Lam H-M (2012). Genome-wide characterization of nonreference transposons reveals evolutionary propensities of transposons in soybean. Plant Cell.

[32] Fang C, Ma Y, Yuan L, Wang Z, Yang R, Zhou Z, Liu T, Tian Z (2016). Chloroplast DNA underwent independent selection from nuclear genes during soybean domestication and improvement. J Genet Genomics.

[33] Guo F, Xiu Z-L, Liang Z-X (2012). Synthesis of biodiesel from acidified soybean soapstock using a lignin-derived carbonaceous catalyst. Appl Energy.

[34] Bandillo NB, Anderson JE, Kantar MB, Stupar RM, Specht JE, Graef GL, Lorenz AJ (2017). Dissecting the genetic basis of local adaptation in soybean. Sci Rep.

[35] Tian Z, Wang X, Lee R, Li Y, Specht JE, Nelson RL, McClean PE, Qiu L, Ma J (2010). Artificial selection for determinate growth habit in soybean. Proc Natl Acad Sci U S A.

[36] Myers N, Mittermeier RA, Mittermeier CG, Da Fonseca GA, Kent J (2000). Biodiversity hotspots for conservation priorities. Nature.

[37] Li YH, Li W, Zhang C, Yang L, Chang RZ, Gaut BS, Qiu LJ (2010). Genetic diversity in domesticated soybean (Glycine max) and its wild progenitor (*Glycine soja*) for simple sequence repeat and single-nucleotide polymorphism loci. New Phytol.

[38] Gordon A, Hannon G (2010). Fastx-toolkit. FASTQ/A short-reads pre-processing tools.

[39] Li H, Durbin R (2009). Fast and accurate short read alignment with Burrows–Wheeler transform. Bioinformatics.

[40] Li H, Handsaker B, Wysoker A, Fennell T, Ruan J, Homer N, Marth G, Abecasis G, Durbin R (2009). The sequence alignment/map format and SAMtools. Bioinformatics.

[41] McKenna A, Hanna M, Banks E, Sivachenko A, Cibulskis K, Kernytsky A, Garimella K, Altshuler D, Gabriel S, Daly M (2010). The Genome Analysis Toolkit: a MapReduce framework for analyzing next-generation DNA sequencing data. Genome Res.

[42] Wysoker A, Tibbetts K, Fennell T. Picard tools version 1.90. 2013.

[43] Danecek P, Auton A, Abecasis G, Albers CA, Banks E, DePristo MA, Handsaker RE, Lunter G, Marth GT, Sherry ST (2011). The variant call format and VCFtools. Bioinformatics.

[44] Browning SR, Browning BL (2010). High-resolution detection of identity by descent in unrelated individuals. Am J Hum Genet.

[45] Krzywinski M, Schein J, Birol I, Connors J, Gascoyne R, Horsman D, Jones SJ, Marra MA (2009). Circos: an information aesthetic for comparative genomics. Genome Res.

[46] Durand EY, Patterson N, Reich D, Slatkin M (2011). Testing for ancient admixture between closely related populations. Mol Biol Evol.

[47] Zheng Y, Janke A (2018). Gene flow analysis method, the D-statistic, is robust in a wide parameter space. BMC Bioinformatics.

[48] Yang Z (1997). PAML: a program package for phylogenetic analysis by maximum likelihood. Bioinformatics.

[49] Kumar S, Stecher G, Tamura K (2016). MEGA7: molecular evolutionary genetics analysis version 7.0 for bigger datasets. Mol Biol Evol.

[50] He Z, Zhang H, Gao S, Lercher MJ, Chen W-H, Hu S (2016). Evolview v2: an online visualization and management tool for customized and annotated phylogenetic trees. Nucleic Acids Res.

[51] Development Core Team R (2011). R: a language and environment for statistical computing.

