# Supplementary material for: Genomic introgression through interspecific hybridization counteracts genetic bottleneck during soybean domestication
Source: Genome Biol. 2019 Jan 30;20:22. doi: 10.1186/s13059-019-1631-5 (PMC6354408; doi:10.1186/s13059-019-1631-5)
Supplement: Supplementary file 2 — Figure S1. Proportions of introgressed fragments in each of the G. soja and G. max accessions investigated. Figure S2. The statistics of introgression frequency in each window among 22 selected accessions. Figure S3. Genome-wide distribution of introgressed G. max fragments in a G. soja accession PI 578357. Figure S4. Haplotypes surrounding the GmHs1-1 region. Figure S5. Haplotypes surrounding the B1 region. Figure S6. Asymmetric divergence of the nuclear and chloroplast genomes within the G. soja or G. max subpopulation. Figure S7. Distribution of divergence time between any two accessions (DOCX 17 MB). [file 13059_2019_1631_MOESM2_ESM.doc]

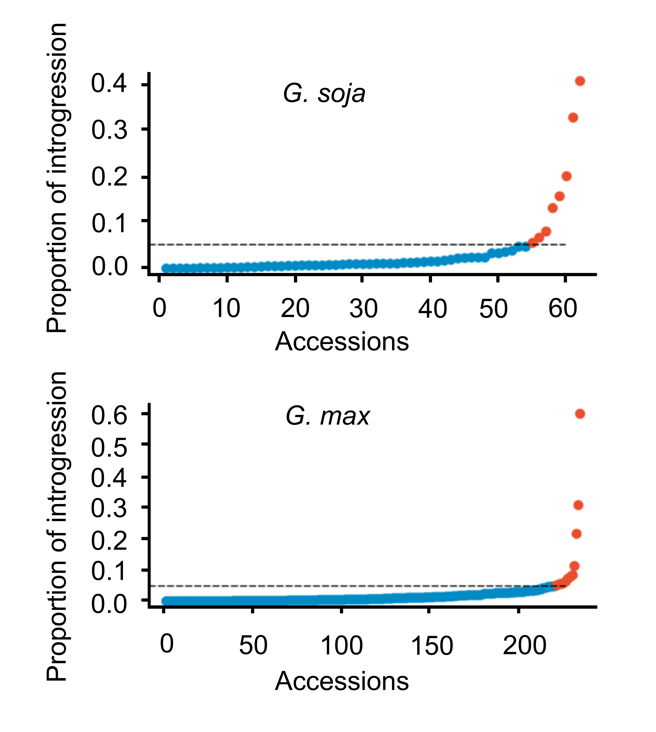


**Figure S1.** **Proportions of introgressed fragments in each of the *G. soja* and *G. max* accessions investigated.**  Individual accessions each harboring >5% introgression were indicated by red dots, while individual accessions each with <5% introgression were indicated by blue dots.

**
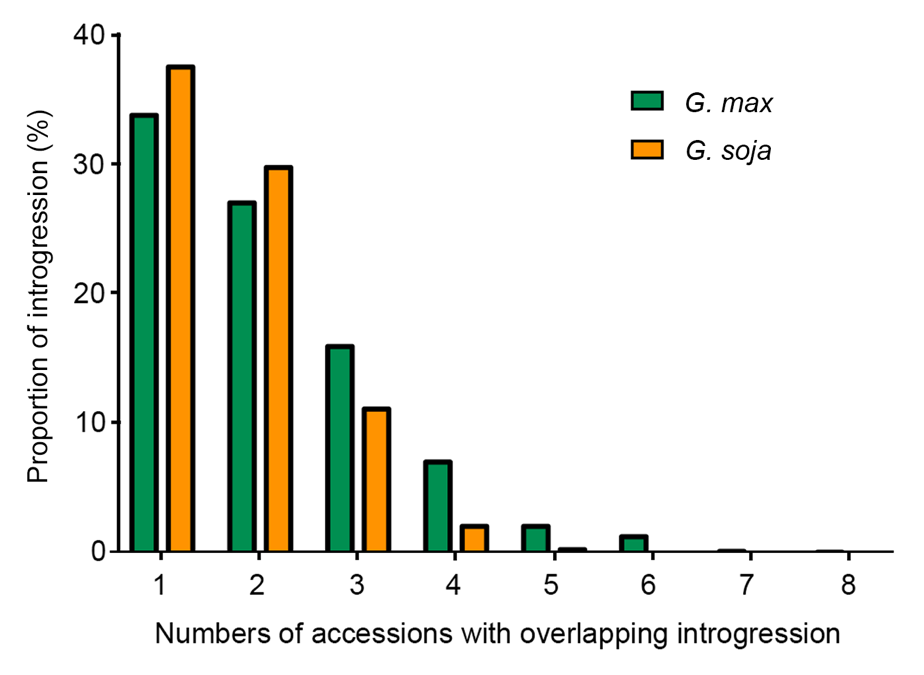
**

**Figure S2.** **Proportions of overlapped introgression among 22 selected accessions.**


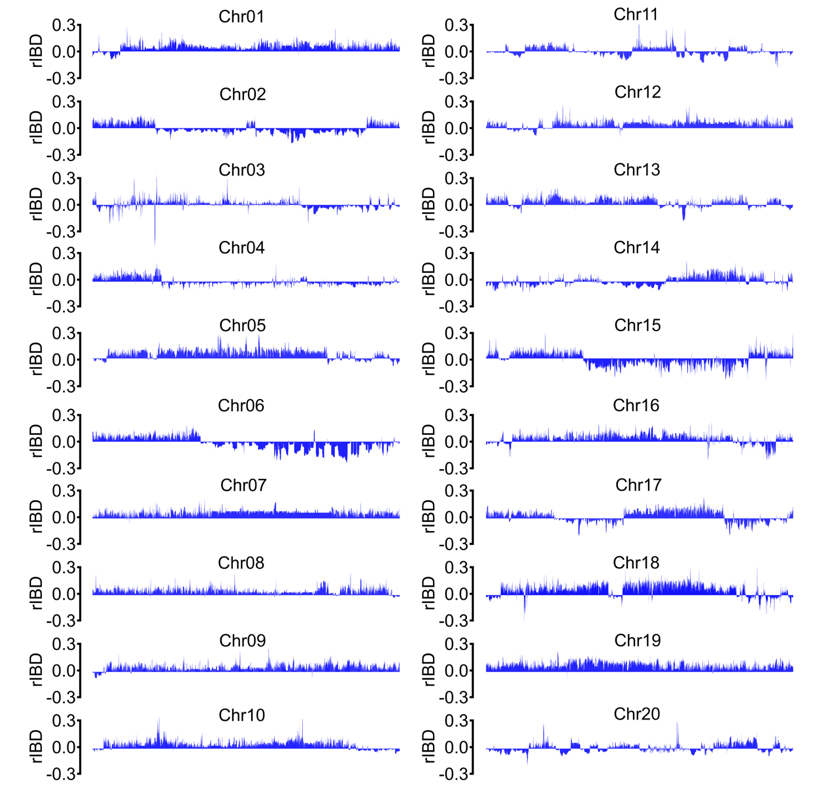


**Figure S3.** **Genome-wide distribution of introgressed *G. max* fragments in a *G. soja* accession PI 578357.** The *x*-axes show chromosomes and the *y*-axes represent relative Identical by Descent (rIBD) that reflect genomic introgression.


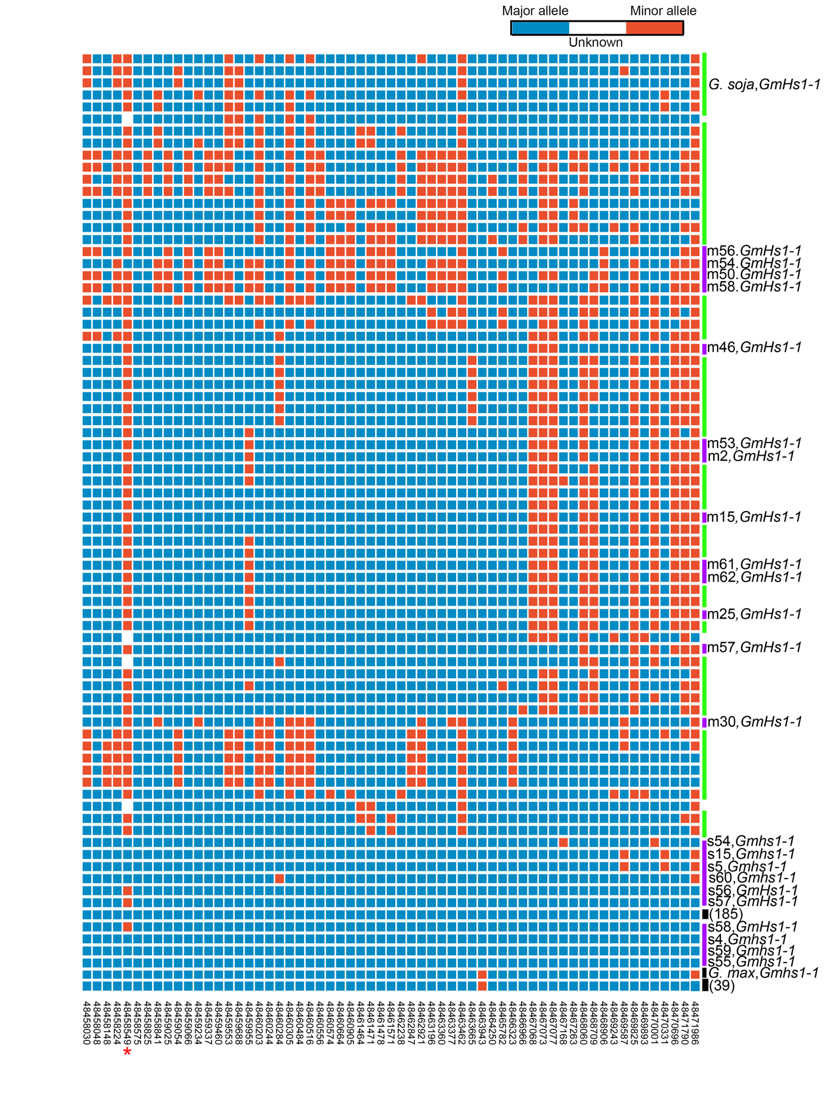


**Figure S4.** **Haplotypes surrounding the *GmHs1-1* region.** The blue color indicates major frequency alleles at each SNP site, while the red color indicates minor frequency alleles. The black bars on the right represent *G. max* accessions, the light green bars on the right represent *G. soja* accessions, the purple bars on the right indicate wild accessions with domesticated alleles or domesticated accessions with the wild-type alleles, and the thick bars show the numbers of accessions sharing the same haplotype. *GmHs1-1* and *Gmhs1-1* are the two alleles of the locus controlling hard seededness.. The red star indicates the causal mutation at the *GmHs1-1* locus.


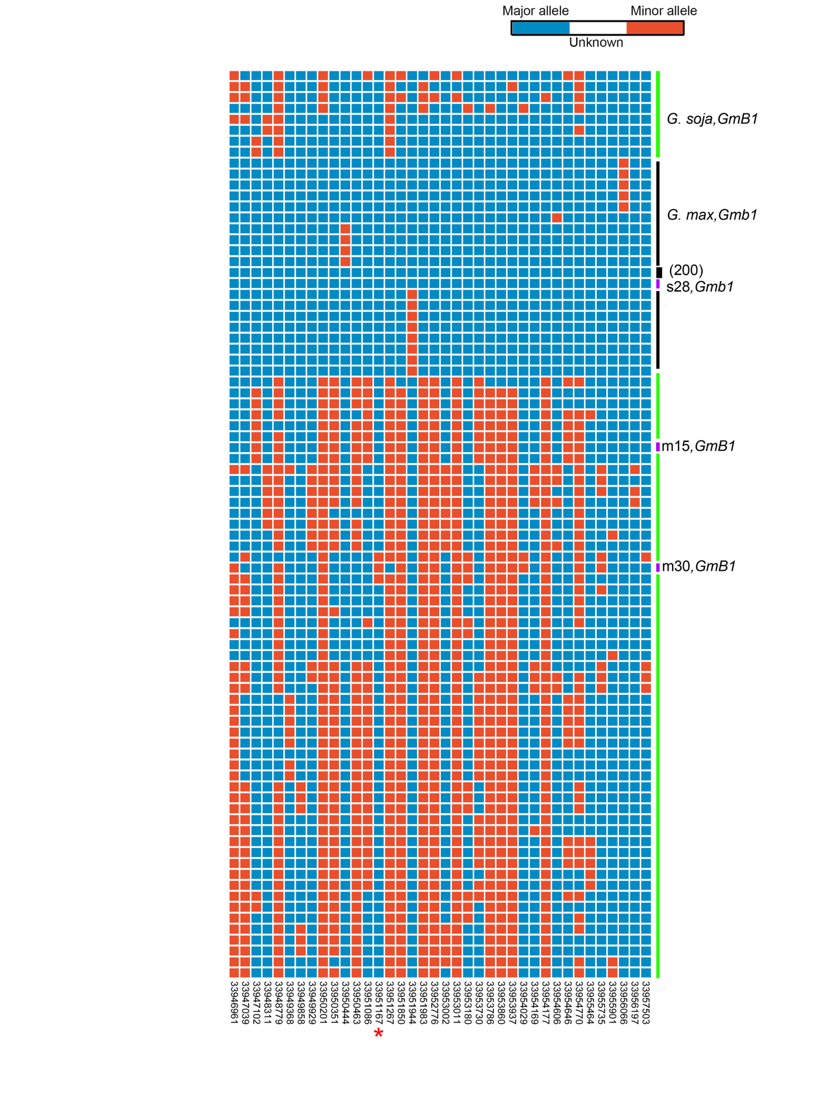


**Figure S5.** **Haplotypes surrounding the *B1* region.** The blue color indicates major frequency alleles at each SNP site, while the red color indicates minor frequency alleles. The black bars on the right represent *G. max* accessions; the light green bars on the right represent *G. soja* accessions, the purple bars on the right indicate wild accessions with domesticated alleles or domesticated accessions with the wild-type alleles. The thick bar on the right shows the number of accessions sharing the same haplotype. *B1* and *b1* are the two alleles of the locus controlling seed coat bloom. The red star indicates the causal mutation at the *B1* locus.


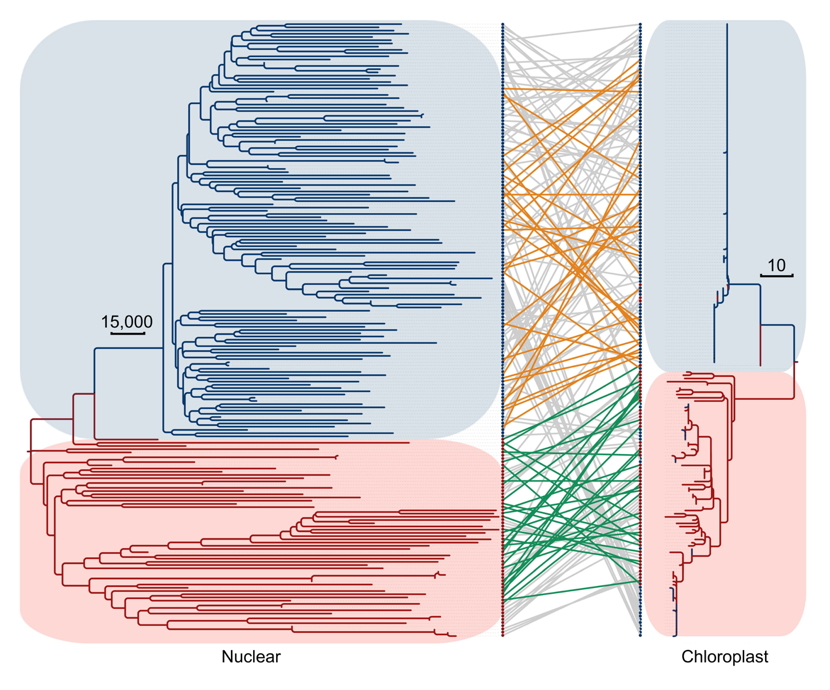


**Figure S6. Asymmetric divergence of the nuclear and chloroplast genomes within the *G. soja* or *G. max* subpopulation.** The phylogenetic tree of the nuclear genomes of the 191 accessions were constructed using all SNPs detected in the whole genome, while the phylogenetic tree of the chloroplast of the same set of accessions were constructed using 333 highly confident SNPs distributed across the chloroplast genome. The *G. max* accessions were indicated by blue branches of two trees and marked by blue dots while the *G. soja* accessions were indicated by red branches of two trees and marked by red dots. The same accessions in the two trees were connected by lines. The green lines indicate *G. max* accessions possessing the *G. soja*-type chloroplasts, and the orange lines indicate the *G. max* accessions showing asymmetric divergence with their *G. max*-type chloroplasts.


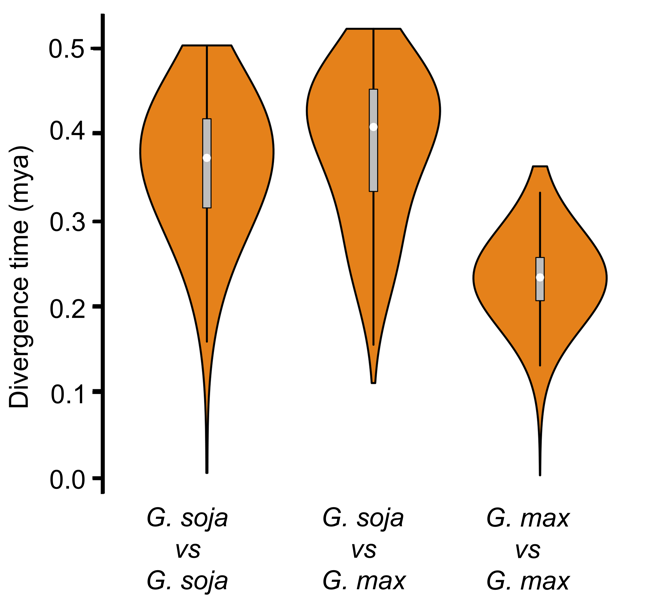


**Figure S7.** **Distribution of divergence time between any two accessions between or within the investigated *G. soja* and *G. max* subpopulations**.
